# Relationship between calcium-to-magnesium ratio and malaria parasite density among children with uncomplicated malaria infection

**DOI:** 10.4314/ahs.v24i2.18

**Published:** 2024-06

**Authors:** Oziegbe Johnson Airen, Loveth Amenaghawon Emokpae, Zainab Omoruyi, Mathias Abiodun Emokpae

**Affiliations:** Department of Medical Laboratory Science, School of Basic Medical Sciences, College of Medical Sciences, University of Benin, Benin City, Edo State, Nigeria

**Keywords:** Child, calcium, magnesium, parasites

## Abstract

**Background/Objective:**

A high calcium-to-magnesium ratio above 2:1 has been associated with higher risk of metabolic, inflammation and cardiovascular disorders. This study evaluates the serum levels of iron, magnesium, calcium, folate, vitamin B12 and calcium to magnesium ratio in children with uncomplicated malaria infection.

**Materials and Methods:**

Measured nutritional parameters were determined in 300 children (100 males and 100 females) with malaria infection and 100 children (50 males and 50 females) without malaria infection using Enzyme linked Immunosorbent Assay and spectrophotometric methods.

**Results:**

Significantly lower (p<0.001) levels of serum magnesium, iron, vitamin B12, folate and Packed cell volume (p<0.03) were observed among children with malaria than controls. On the other hand, serum calcium (8.45±0.20) and calcium-to-magnesium ratio (3.9:1.0) (were significantly higher (p<0.001) in malaria infected children than controls. Calcium to magnesium ratio correlated (r=0.188; p<0.01) with malaria parasitaemia.

**Conclusion:**

Higher serum calcium-to-magnesium ratio above the recommended 2.1 may contribute to increase risk of morbidity and mortality. Nutritional intervention aimed at lowering the ca/mg ratio may be essential in the management of malaria infection in Children.

## Introduction

Malaria infection is still a leading cause of death and hospitalization among children in Nigeria and other countries in sub-Saharan Africa. The causative organisms are by malaria parasites (Plasmodium species) which are transmitted by the female anopheles mosquito (vector). Epidemiological studies have shown that *P. falciparum* is the most dangerous specie and it is the cause of most of the deaths caused by malaria infection 2. Despite several measues employed to eliminate or reduce the infection especially among children and pregnant women, mortality due to malaria infection has not abated and malaria infection is the major reason for medical consultation in most outpatients clinics in Nigeria^3^.

An adequate nutritional status is an important factor that reduces disease progression and improves survival from illnesses^4^. One of the most important risk factors for nutritional deficiencies is malaria infection which may be attributable to a decreased nutrient intake, especially during periods of acute illness and disease progression/severity. Also, subjects with malaria infection may have higher than normal requirements for macro and micro-nutrients such as calcium (Ca), magnesium (Mg), folate and vitamin B_12_ due to the increased sweating, intestinal malabsorption, and chronic inflammation that are common in malaria infection^5^. However, an imbalance in calcium-to- magnesium ratio in malaria infected subjects is under-recognized^6^, and the analytes are rarely investigated routinely.

Insufficient dietary intake of nutrients such as calcium and magnesium may be associated with increased risk of malaria, cardiovascular diseases, metabolic diseases and mortality^7^. Magnesium is second to calcium as the most abundant divalent cations in the body and both elements can potentially antagonize each other in physiologic systems. Some authors have shown that circulating calcium and magnesium levels may be influenced by malaria parasite infection^8^ and potentially cause increase inflammation, cardiovascular disorders and mortality. Therefore, inadequate dietary calcium and magnesium, or an imbalance between the two, may have adverse effects on overall health and mortality^9^. Even though the study that assessed the association between calcium to magnesium ratio in children with malaria infection is scarce in our setting, the evidence for the importance of calcium to magnesium ratios in overall health is on the increase^10^-^12^. Magnesium deficiency may lead to fatigue, vomiting, weakness, muscle contraction and cramps. The unique biologic roles of magnesium make it an essential cation that is needed in relatively large amounts in humans^13^. Magnesium also acts as a cofactor in several enzymatic reactions essential for human physiological functions which include heart beats, vascular tone, nerve function, muscle contraction and relaxation^13^.

Calcium provides strength for bone and teeth and plays essential roles in the maintenance of health as well as nutritional qualities^14^.

Iron (Fe) is an essential micronutrient and component of red blood cell which is involved in the transportation of oxygen from the lungs to the various tissues in the blood^15^. It is also a component part of some enzymes responsible for peroxides and cytochromes activities involved in cellular and mitochondrial respiratory mechanisms^16^.

Conflicting reports exist in literature as to the levels of iron, magnesium and calcium among individuals infected with malaria. Whereas some authors have reported significantly higher levels of these nutrients others observed significantly lower levels compared with controls 1,17,18. Since high calcium to magnesium ratio has been linked with all-cause and cardiovascular mortality in several diseases, this study seeks to evaluate the levels of packed cell volume, serum iron, magnesium, calcium, folate and vitamin B^12^ in children with uncomplicated malaria infection. It also determines the association between calciumto- magnesium ratio and malaria parasite density among children with *P. falciparum* infection.

## Materials and methods

**Study Design and location:** This is a cross sectional study of children suffering from malaria infection in Central Hospital, Benin City. They consist of 200 children (100 males and 100 females) with malaria infection and 100 children (50 males and 50 females) without malaria infection which served as controls. Sixty participants with malaria infection and 40 without malaria infection age range 6months to 4years; 71 cases and 40 control subjects' age range 5years to 10years and 69 cases and 20 control subjects age range 10 to 12 years were enrolled in the study.

### Inclusion and exclusion criteria

Children within the ages of 6months and 12 years who were confirmed to be suffering from malaria infection by the presence of malaria parasite and whose parents or guardians gave informed consent for their wards to participate in the study were recruited. The children who were on anti-malaria medication, micronutrients supplementation and whose parents or guardians refused to give inform consent to participant in the study were excluded. The control group was apparently healthy children without evidence of malaria infection by microscopic examination of blood smears. Those known to be suffering from sickle cell disease were also excluded.

### Ethical consideration

The study protocol was obtained from the Edo State Ministry of Health (CH/A406 VOLIII/195 dated 3rd June 2015). All subjects who gave informed consent and met the inclusion criteria were enrolled into the study.

### Sample Size determination

#### The minimum sample size was determined using the sample size determination

formula for health studies^18^. A prevalence of 8.6% of malaria among Children in the Niger Delta Region of Nigeria19 was used for this study. N = Z2 × P (1 − P)/d2 where n = minimum sample size, P = estimated prevalence, Z = standard normal deviate that corresponds to 95% confidence limit (1.96) and 1 is the alpha level of significance (5%). Using this formula, the calculated sample size was 121, but 200 subjects was recruited for this study.

#### Sample Collection

Five milliliters of venous blood was collected with 2mL dispensed into EDTA container while 3mL was emptied into plain plastic container and was allowed to clot at room temperature. This was centrifuged at 3000 rpm for 10minutes. The serum was separated into another plain plastic container and was kept frozen at -20°C until analyses were done. The blood specimen collected in EDTA was used for thin and thick blood films to detect malaria parasites.

### Laboratory Analysis

#### Thick Film for Malaria Parasite

Using a grease-free microscope slide, a large drop of blood about 15mm was placed on the slide. Without delay, the end of a plastic bulb pipette was used to spread the drop of blood to make the thick smear which covers an area of 15 x 15mm. The blood films were then allowed to dry. The dried thick blood smear was stained and examined using Giemsa stain and light microscopy respectively. Smears were then examined using X100 oil immersion. At least 100 high power fields were examined before a thick smear was declared negative. Plasmodium falciparum parasites were counted per 200 or 500 leukocytes, which were used to determine the parasite density per microlitre of blood. Thin films were examined to confirm the species identification on the thick film. The malaria parasites were enumerated and reported thus: Low parasitaemia, 1-10(+), moderate parasitaemia, 11-29(++) and high parasitaemia, >30(+++)/x100 high power field.

#### Assay of measured nutrients

Serum iron, calcium and magnesium were determined by colorimetric method using reagents supplied by TECO diagnostic, Anaheim, CA, USA while vitamin B12 and folic acid were evaluated by ELISA technique using reagents supplied by Elabscience Biotechnology, Houston, Texas, USA. In order to ensure accuracy and precision of results, quality control sera supplied by Bio-Labo, France were included in the assay.

The principles of measured biomarkers are as follows: Serum calcium at mildly acidic pH, metallo-chromogen Arsenazo III combines with calcium to form a coloured complex whose absorbance is measured at 650nm. The intensity of the absorbance is proportional to the amount of calcium in the sample.

Magnesium reacts with xylidyl-blue to form a coloured compound in alkaline solution which is read colorimetrically.

Serum iron III reacts with chromoazural B (CAB) and cetyltrimethyl ammonium bromide (CTMA) to form a coloured ternary complex whose absorbance is read at 623nm. The intensity of the colour produced is directly proportional to the concentration of iron in the sample. Serum vitamin B12 and folic acid were determined using ELISA technique; Electroluminescence is a competitive inhibition enzyme immunoassay technique using a monoclonal antibody specific to vitamin B12 or folic acid pre-coated onto a microplate. The end product of substrate hydrolysis is inversely proportional to the concentration of vitamin B12 or folic acid in the sample.

#### Statistical Analysis

The results obtained were analyzed using a statistical software package (SPSS version 20, IBM, IL, USA). Students't-test was used to compare the levels of measured variables between malaria infected subjects and controls. Calcium-to-magnesium ratio was correlated with malaria parasite density using Pearson correlation coefficient and a p-value of ≤0.05 was considered significant.

## Results

The characteristics of study participants and malaria parasite count are presented in table 1. The age of the subjects' ranges from 6 months to 12 years with a mean of 8.26±1.2 and the malaria parasite count varies from 1+ to 3+ among the children with malaria infection. The levels of serum magnesium (2.13±0.07), iron (102.12±1.96), vitamin B12 (435.53±43.00), folic acid (7.28±0.20) and PCV (31.5±0.10) were significantly lower (p<0.05) among children with malaria infection compared with control subjects. Conversely, serum calcium (8.45±0.20) and calcium-to-magnesium ratio (2.13±0.07) were significantly higher (p<0.001) in malaria infected children when compared to control subjects (table 2). Calcium-to-magnesium ratios increased with increasing malaria parasite densities. The Ca/Mg ratio was 2.8:1.0 at low parasitaemia (<10), 3.1:1.0 at moderate parasitaemia (11-29) and 4.2:1.0 at high parasitaemia (>30) and Ca/Mg ratios correlated positively with parasite densities (0.188,p<0.01) (table 3).

**Table 1:** Characteristics of study participants and Malaria parasite count

Variables	Study Participants(n=200)	Controls (n=100)
**Age brackets**		
**6moths – 4Years**	60	40
**5-10 Years**	71	40
**10-12Years**	69	20
**Gender**		
**Males**	100	50
**Females**	100	50
**Malaria Parasite Count**		
**(1+)**	50	-
**(2+)**	80	-
**(3+)**	70	-

**Table 2:** Comparison of measured variables between malaria infected and non-malaria infected children

Measured Variables	Malaria infected Children	Non-Malaria infected children	P-Value
**Calcium (mg/dL)**	8.45±0.20	12.26±0.30	0.001
**Magnesium(mg/dL)**	2.13±0.07	5.85±0.30	0.001
**Iron(mg/dL)**	102.12±1.96	167.26±3.0	0.001
**VitaminB12(pmol/L)**	435.53±43.00	772.31±57.12	0.001
**Folic acid(µg/L)**	7.28±0.20	17.67±0.50	0.001
**Calcium:magnesium ratio**	3.9±0.03	2.0±0.02	0.001
**Packed cell volume (%)**	31.5±0.10	36.8±0.10	0.03

**Table 3:** Stratified Analyses of calcium-to-magnesium Ratio based on Malaria Parasite Density

Malaria Parasite Density	Calcium/Magnesium Ratio	R-Value	P-Value
Low parasitaemia (1-10/µL)	2.8±0.02		
Moderate parasitaemia (11-29/µL)	3.1±0.03	0.188	0.01
High parasitaemia (>30/µL)	4.2±0.03		

## Discussion

It was reported that significant changes of some macro and micro-nutrients do occur in malaria infection^1^, a condition that may be worrisome in sub-Saharan Africa where malnutrition and malaria infection co-exist. The need for adequate documentation of such findings by constant surveillance cannot be our-emphasized, since progression of mild to severe deficiencies in children with malaria may be rapid. In this study, serum magnesium, iron, vitamin B_12_ and folic acid levels were significantly lower (p<0.001) while calcium and calcium-to-magnesium ratio were significantly higher (p<0.001) in children with malaria infection than malaria negative subjects. The finding is consistent with previous studies^21^-^23^. The authors stated that alteration in the level of calcium-to-magnesium ratio caused by *P. falciparium* infection may be linked with certain errors of metabolism leading to morbidity and mortality among children. The prevention of induced depletion of these nutrients with micronutrients supplementation has been suggested^24^. Low serum magnesium concentration and higher calcium-to-magnesium ratio may impair DNA repair, cell differentiation and proliferation, increase apoptosis and angiogenesis as well as inflammatory response and oxidative stress^21^,^23^. Since magnesium is involved in several physiological activities, deficiency may affect multiple pathways leading to increased susceptibility and/or pathogenesis of malaria infection^21^. Significantly higher (p<0.05) serum levels of copper and magnesium was reported among children with uncomplicated malaria infection^17^.

The result of this study also shows that calcium-to-magnesium ratio is associated with malaria density in malaria infected children. High calcium-to-magnesium ratio correlated with poor outcome of cardiovascular mortality in many diseases. Some authors also reported that high calcium-to-magnesium ratio was significantly linked to all-cause and cardiovascular mortality in patients on dialysis^25^. It is suggested that magnesium deficiency and high calcium-to-magnesium ratio may have negative effect on the endothelial function leading to cardiovascular events in malaria infection. High calcium-to-magnesium ratio has been associated with traditional markers of cardiovascular risk and inflammation such as troponin^26^, neutrophil-to-lymphocyte ratio^27^ and c-reactive proteins^28^ by several authors. This finding aligned with that of Kamal and Ahmed8, who reported that serum Mg levels were significantly declined with increasing parasitaemia from low parasitaemia, moderate parasitaemia and high parasitaemia among Sudanese subjects with malaria infection. Conversely, the finding from this study did not agree with some other studies 29,30. The authors reported lower levels of calcium in subjects with malaria infection. They opined that the lower concentrations of calcium may be due to the accumulation of calcium in the intestinal compartment of trophozoites for metabolism, loses during digestive and renal perturbation during the course of malaria infection and the decreased intestinal absorption of calcium.

A study from Tanzania shown that iron deficiency may be protective against malaria infection and deaths in children hence risk of malaria infection may be influenced by physiologic status of iron^30^. An insignificant change in the level of iron between malaria infected young adults and controls was reported by these authors^17^.

Imbalance in calcium-to-magnesium ratio may result in inflammation, oxidative stress and pathogenic sequelae of malaria infection in children. The reference range of calcium-to-magnesium ratio is not available locally in children but a ratio of 1.7 to 2.63 was required for high intake of calcium and magnesium in order to protect against colorectal cancer^24^. Dean6 suggested that calcium-to-magnesium imbalance above the recommended 2.1 may exacerbates the risk of metabolic, inflammatory and cardiovascular disorders in children. Low magnesium levels may cause heart disorders, pains, anxiety, abdominal discomfort and diabetes.

Serum folate, vitamin B and iron were significantly higher (p<0.001) in malaria infected children than non-malaria infected control subjects. This finding is consistent with previous studies^31^,^32^. Folate, vitamin B_12_ and iron deficiencies often result in megaloblastic anaemia. Also, the metabolism of folate and vitamin B_12_ is inter-related and deficiency in either vitamin may lead to megaloblastic anaemia. Folate deficiency has been associated with impaired cognitive development, diarrhea and respiratory disease^33^. Folate functions in single carbon transfers in the formation of DNA and RNA as well as in the metabolism of amino acids. Major folate dependent reactions include the conversion of homocysteine to methionine and the conversion of deoxyuridylate to thymidylate which is necessary for cell division. A deficiency of folate leads to a rise in homocysteine levels and impairs the synthesis of red blood cells which manifests as megaloblastic anaemia^33^.

Routine supplementation of these micronutrients has been recommended as an anaemia control strategy among children in regions where anaemia is common^34^. However, it was suggested that supplemental folate should be used with caution in infants and young children in countries where anti-folate anti-malaria drugs are prescribed. This is to guide against the potential interference of folate with anti-folate anti-malaria drugs. Some authors have suggested that high folate levels may favour the growth of *P. falciparum* and slows or inhibits parasite clearance during treatment with anti-folate anti-malaria drugs, since folate is required by the malaria parasite for survival. The parasite's folate metabolism is an important target for anti-folate drugs that are folate antagonists. Examples of such drugs include sulphadoxine-pyrimethamine (Fansidar), pyrimethamine-sulphalene (Metakelfin), atovaquone-proguanil (Malarone), pyrimethamine-dapsone (Maloprim). These anti-malaria drugs target the enzymes dihydrofolate reductase and dihydropteroate synthase, which are needed in folate pathway^35^,^36^. Therefore, these drugs should be used with caution in subjects who are already folate deficient^36^.

The role of folate in the pathogenesis of malaria infection from animal and observational human studies has been controversial. Whereas folate deficiency in rhesus monkeys was protective against malaria infection, folate deficiency among chickens exacerbated malaria infection^33^. In the same vein, in-vitro studies have shown that the addition of folate to test media inhibited the action of anti-folate anti-malaria drugs^37^,^38^. Megaloblastic anaemia due to folate and vitamin B12 deficiencies was associated with severity of malaria infection among children in Nigeria^39^. The study indicates that folate deficiency may lead to higher susceptibility to malaria infection in children. Absence of folate deficiency and megaloblastic anaemia was suggested to be responsible for low malaria infection rates among pregnant women in Uganda^40^. Children with P.falciparum malaria infection in Sudan were reported to have significantly higher levels of homocysteine than control subjects^32^. The observation shows that deficiencies of vitamin B12 and folate may cause increased risk of malaria infection. It should be noted that none of these studies were able to establish whether the deficiencies was the cause of malaria infection.

## Conclusions

Higher serum calcium-to-magnesium ratio above the recommended 2.1 was observed in this study and may contribute to increase risk of morbidity and mortality. Nutritional intervention aimed at lowering the calcium to magnesium ratio may be necessary in the management of malaria infection in children.
